# Effects of Poly(Amidoamine) Dendrimer-Coated Mesoporous Bioactive Glass Nanoparticles on Dentin Remineralization

**DOI:** 10.3390/nano9040591

**Published:** 2019-04-10

**Authors:** Jungin Bae, Woo-Sung Son, Kyung-Hyeon Yoo, Seog-Young Yoon, Moon-Kyoung Bae, Dong Joon Lee, Ching-Chang Ko, Youn-Kyung Choi, Yong-Il Kim

**Affiliations:** 1 Department of Orthodontics, Dental Research Institute, Pusan National University Dental Hospital, Yangsan 50612, Korea; jib19@naver.com (J.B.); wsson@pusan.ac.kr (W.-S.S.); 2 School of Materials Science and Engineering, Pusan National University, Busan 46241, Korea; seweet07@pusan.ac.kr (K.-H.Y.); syy3@pusan.ac.kr (S.-Y.Y.); 3 Department of Oral Physiology, School of Dentistry, Pusan National University, Yangsan 50612, Korea; mkbae@pusan.ac.kr; 4 Oral and Craniofacial Health Sciences Research, School of Dentistry, University of North Carolina at Chapel Hill, Chapel Hill, NC 27516, USA; dongjoon_lee@unc.edu; 5 Department of Orthodontics, School of Dentistry, University of North Carolina at Chapel Hill, Chapel Hill, NC 27516, USA; ching-chang_ko@unc.edu; 6 Department of Orthodontics, Biomedical Research Institute, Pusan National University Hospital, Busan 49241, Korea; 7 Institute of Translational Dental Sciences, School of Dentistry, Pusan National University, Busan 49241, Korea

**Keywords:** PAMAM, mesoporous bioactive glass, dentin hypersensitivity, dentinal tubule occlusion

## Abstract

Dentin hypersensitivity (DH) is one of the most common clinical conditions usually associated with exposed dentinal surfaces. In this study, we identified the effectiveness of poly(amidoamine) (PAMAM) dendrimer-coated mesoporous bioactive glass nanoparticles (MBN) (PAMAM@MBN) on DH treatment, examining the ion-releasing effect, dentin remineralization, and the occluding effect of dentinal tubules. We synthesized MBN and PAMAM@MBN. After soaking each sample in simulated body fluid (SBF), we observed ion-releasing effects and dentin remineralization effects for 30 days. Also, we prepared 30 premolars to find the ratio of occluded dentinal tubules after applying MBN and PAMAM@MBN, respectively. The results showed that PAMAM did not disrupt the calcium ion-releasing ability or the dentin remineralization of MBN. The PAMAM@MBN showed a better occluding effect for dentinal tubules than that of MBN (*p* < 0.05). In terms of dentinal tubule occlusion, the gap between MBN was well occluded due to PAMAM. This implies that PAMAM@MBN could be effectively used in dentinal tubule sealing and remineralization.

## 1. Introduction

Dentin hypersensitivity (DH) is one of the most common clinical conditions usually associated with exposed dentinal surfaces. According to the widely accepted hydrodynamic theory, the fluid within the dentinal tubules, absent of a smear layer, is subject to thermal, chemical, tactile or evaporative stimuli. The movement of the fluid within the dentinal tubules stimulates the mechanical receptors which are sensitive to fluid pressure, resulting in the transmission of the stimuli to the pulpal nerves, ultimately causing pain response [1]. Dentin hypersensitivity is caused by the exposure of the dentinal tubule, and with the increasing diameter of the tubule, dentin is demineralized by micro-organisms within the oral cavity or by acidic food [2,3,4]. Thus, the key point of DH treatment is to desensitize dentin through the remineralization of exposed dentin-occluding dentinal tubules [5,6].

Many kinds of studies have been done to identify dentin-desensitizing materials, and it has been found that sodium fluoride solution [7], calcium hydroxide, stannous hydroxide, calcium oxalate, ferric phosphate [8], and potassium nitrate solution [9] are effective for dentine hypersensitivity treatment. Also, dental products like fluoride varnishes [10] and resin adhesives [11] are proven to be helpful in decreasing DH, by occluding dentinal tubules. Low-level laser treatment, such as the Nd:YAG laser [12], has potential for DH treatment. Recently, bioactive glass (BAG) has been proposed as a good option for DH treatment, due to its excellent remineralization ability and bioactivity [13].

Bioactive glass is a material that is composed of SiO_2_–CaO–Na_2_O–P_2_O_5_, which has been proven to be effective in occluding dentinal tubules and for dental remineralization [14,15,16,17,18,19]. Bioactive glass decomposes calcium and phosphorus ions within the environment of the oral cavity, and it forms hydroxycarbonate apatite (HCA), which helps with the occlusion of dentinal tubules and dental remineralization [15,16,20,21,22].

A dendrimer is a macromolecule with a branch-shaped unit structure that repeatedly spreads from the core molecule. In three dimensions, its structure is nearly spherical, and although its core has a relatively low density, the further it spreads from the core, the higher the density of branches. The dendrimer macromolecule is well defined in its structure, and it is easy to form nano-sized particles by predicting and synthesizing exact molecular weights and structures. The end-part that is located in the most outer part of the dendrimer is known to have a critical influence on the surface properties and solubilities of the dendrimer, and it is also possible to introduce functional groups and various derivatives at the dense end-parts of the surface [23,24,25]. Due to these features, it is used as a scaffold for nanoparticles in various clinical fields, such as drug and gene delivery, tumor targeting, and vaccine delivery [26,27,28].

Poly(amidoamine) (PAMAM), one of these dendrimers, is a highly developed polymer with reactive end-parts in various forms and sizes inside the sphere [29]. Poly(amidoamine) is a notable dendrimer in biomineralization fields, because it can perform in a similar manner to non-collagenous proteins (NCPs) that play an important role in dentin mineralization [29,30,31].

Also, with its relatively lower toxicity, better biocompatibility, and wider surface area, it is considered as potentially providing an ideal platform for hydroxyapatite reformation [32].

According to Tao S. et al., PAMAM–NH_2_ reacts with calcium and phosphorus ions to form hydroxyapatite, and it has been experimentally verified that it could be effective in dentin remineralization [33]. In particular, in terms of intrafibrillar remineralization within the dentinal tubule, studies on the remineralization effect of PAMAM showed quite an interesting result. Furthermore, intrafibrillar remineralization using nanostructures has significance in DH treatment [23].

Studies on dentinal remineralization have shown that BAG and PAMAM could be effective in DH treatment, with their distinctive properties. If these two materials are synthesized into new materials to yield a higher reactivity for PAMAM and the ion-releasing effects of BAG, a better nanostructured biomaterial can be expected in the dentin remineralization field. However, such studies have not yet been conducted.

For the DH treatment, not only is the effective occlusion of dentinal tubules over the long-term and short-term period essential, but their stability toward edible acidic exposure is also important, and intrafibrillar remineralization within dentin is critical in the long term [34,35].

Therefore, the aim of this study is to synthesize PAMAM and mesoporous bioactive glass nanoparticles (MBN) and PAMAM-coated MBN (PAMAM@MBN) in order to compare the ion-releasing effects, dentin remineralization, and occluding effects of dentinal tubules.

## 2. Materials and Methods

### 2.1. Mesoporous Bioactive Glass Nanoparticle (MBN) Synthesis

Mesoporous bioactive glass nanoparticles (MBN) were synthesized with a modified method from Lee et al. [36] and El-Fiqi et al. [37]. In 150 mL distilled water, 2-ethoxyethanol (Sigma-Aldrich, St. Louis, MO, USA), 2 mL aqueous ammonia (Samchun, Pyeongtaek, South Korea), 1.4 g calcium nitrate tetrahydrate (Ca(NO_3_)_2_·4H_2_O) (Sigma-Aldrich), and 20 mL ethanol (Samchun, Pyeongtaek, South Korea) were combined; then, 1 g hexadecyltrimethylammonium bromide (CTAB) (Sigma-Aldrich) was added, and the mixture was stirred for 30 min at room temperature. After adding 5 mL of tetraethyl orthosilicate (TEOS) (Sigma-Aldrich), 4 h of vigorous stirring was performed. The molar ratio (mol %) of the resulting CaO:SiO_2_ was calculated to be 15:85. A white precipitate was obtained, which was washed with ethanol and dried for 24 h at 60 °C. To remove CTAB, calcination was performed for 5 h at a heating rate of 1 °C·min^−1^ at 600 °C.

### 2.2. PAMAM@MBN Synthesis

Poly(amidoamine) dendrimers (ethylenediamine core, generation 4.0 solution) was purchased at Sigma-Aldrich (St. Louis, MO, USA). A total of 0.1 g of PAMAM dendrimer (10 wt % in methane, Sigma-Aldrich) was gradually added with 0.6 g MBN at pH 10~11. Then, overnight stirring was performed at room temperature, followed by sonication for 30 min. The dendrimers were washed with ethanol and dried overnight at 50 °C.

### 2.3. Analysis of Sample Characteristics

The characteristics of the synthesized MBN and PAMAM@MBN were analyzed by using the following methods.

#### 2.3.1. X-Ray Diffraction (XRD) and Fourier Transform Infrared Spectroscopy (FT-IR) Analysis

X-ray diffraction patterns were obtained using an Ultima IV multipurpose XRD system (Rigaku, The Woodland, TX, USA) at 40 kV and 40 mA, with a scanning speed of 0.1°/min. Functionalized surfaces were investigated using the ATR method of FT-IR; specifically, the Spectrum GX FT-IR Spectrometer (PerkinElmer Inc., Waltham, MA, USA) was used.

#### 2.3.2. Surface Area and Pore Size Analysis

Using an adsorption analyzer (Quantachrome autosorb-iQ MP, Boynton, FL, USA) at 77.35 K, N_2_ adsorption–desorption isotherms were obtained. The distribution of the specific surface area and the pore size was measured by the Brunauer–Emmett–Teller (BET) method [38] and the Barrett–Joyner–Halenda (BJH) method [39].

#### 2.3.3. Analytical Field-Emission Scanning Electron Microscopy (FESEM) and Field-Emission Transmission Electron Microscopy (FETEM)

Using FESEM (SU-70, Hitachi, Tokyo, Japan) and a 200 kV field-emission transmission electron microscope (200 KV FETEM) (TALOS F200X, FEI, Hillsboro, OR, USA), sample types were observed.

### 2.4. In Vitro Ion-Releasing Test

To test the ion releasing (Ca, P, Si) of MBN and PAMAM@MBN, MBN and PAMAM@MBN powders were made into a thin plate (5 mm diameter, 2 mm thickness), and placed in simulated body fluid (SBF) after heating the SBF to 36.5 °C. The volume of SBF was calculated by following equation: *Vs*. = *Sa*/10, where *Vs*. is the volume of SBF (mL) and *Sa* is the apparent surface area of specimen (mm^2^) [40]. The entire specimen should be submerged in the SBF. Three samples for each immersion time point were used. After soaking, the ion concentration (Ca, P, Si) was measured for 1, 2, 3, 5, 10, and 30 days, using ICP-OES (Optima 8300, Perkin Elmer, Waltham, MA, USA).

### 2.5. In Vitro Mineralization Ability Test

For apatite-forming ability test, MBN and PAMAM@MBN powders was made into a thin plate (5 mm diameter, 2 mm thickness), and should be placed in SBF after heating the SBF to 36.5 °C. The volume of SBF was calculated by following equation: *Vs*. = *Sa*/10, where *Vs*. is the volume of SBF (mL) and *Sa* is the apparent surface area of specimen (mm^2^) [40]. The entire specimen should be submerged in the SBF. After soaking for 1, 5, and 30 days, the SBF solution was removed, then it was washed three times for five min with distilled water and dried. X-ray diffraction and FT-IR measurements were performed.

### 2.6. Sensitive Tooth Model Preparation

Twenty premolars were collected in a manner that was verified and reviewed by the Institutional Review Board of Pusan National University Dental Hospital (PNUDH-2018-033). The teeth were stored at 4 °C in 0.5% thymol solution, to be used within one month. A one-millimeter-thick dentin disc cut horizontally through the longitudinal axis of the tooth from the downside of the enamel–dentin junction was prepared by using a low-speed diamond saw (Struers Accutom-50, Ballerup, Denmark). The discs were polished with 320- and 600-grit silicon carbide (SiC) polishing paper for 60 s each. Then, they were soaked in 1 wt % citric acid for 20 s and completely rinsed with a water spray, to open the dentinal tubule of the disc. Finally, a sensitive tooth model was made. The specimens were separated into two groups (*n* = 10).

Group 1: 100 mg MBN was applied to the dentin surface at the lower surface areas for 20 s, using a rotary cup that contained a slurry prepared at a ratio against 200 µL deionized water, and applied again for 15 s, for a complete time of 30 s.

Group 2: 100 mg PAMAM@MBN was applied to the entire dentin surface, using the same method mentioned in Group 1, with the slurry prepared at a ratio against 200 µL deionized water.

### 2.7. Field-Emission Scanning Electron Microscopy Assessment of Dentinal Tubule Occlusion

Sensitive tooth model discs applied by MBN or PAMAM@MBN for each group were rinsed with water spray and dried. Afterwards, using FESEM, each treatment was done to observe changes in the exposed dentinal tubule occlusions. The dentinal tubule part of each disc was observed at microscopic magnifications of 2000×, 10,000×, and 40,000 ×. The 2000× image analyzed in Image J (NIH, Frederick, MD, USA) was used to confirm dentin tubule closure, and to calculate the area ratio (the area of occluded tubules/the total tubule area) of the occluded dentinal tubules. The 2000× image could help to calculate a relatively large number of dentinal tubules in an obtained image [41].

### 2.8. Statistical Analysis

All statistical analyses in this study utilized the R language program (version 3.3.2; R Foundation for Statistical Computing, Vienna, Austria). Independent *t*-test was performed for the comparison of dentinal tubule occlusion between MBN and PAMAM@MBN (*p* < 0.05).

## 3. Results

### 3.1. Characteristics of the Samples

#### 3.1.1. XRD Analysis

**Figure 1:**
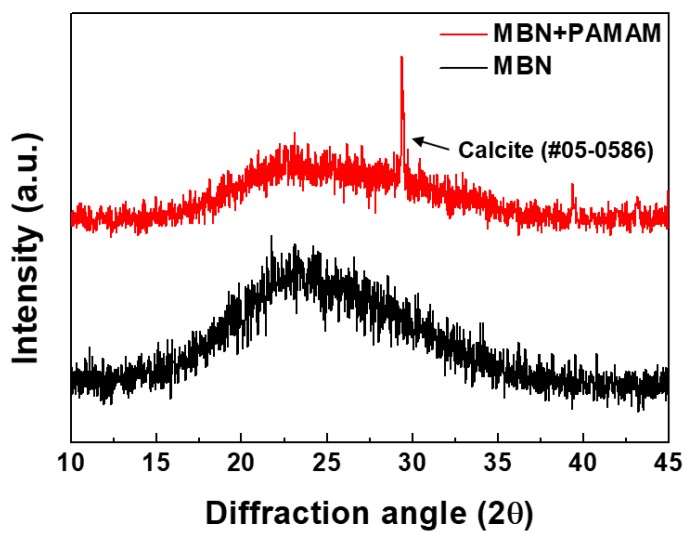
X-ray diffraction (XRD) patterns of mesoporous bioactive glass nanoparticles (MBN) and poly(amidoamine) (PAMAM) dendrimer-coated MBN (PAMAM@MBN).

Figure 1 shows the XRD patterns of MBN and PAMAM@MBN. Based on XRD analysis, MBN has an amorphous form, which means that MBN has a glass-type form. Similar to MBN, PAMAM@MBN has an amorphous form, and showed calcite peak (CaCO_3_, JCPDS #05-0586) at 29.45°.

#### 3.1.2. FT-IR

**Figure 2:**
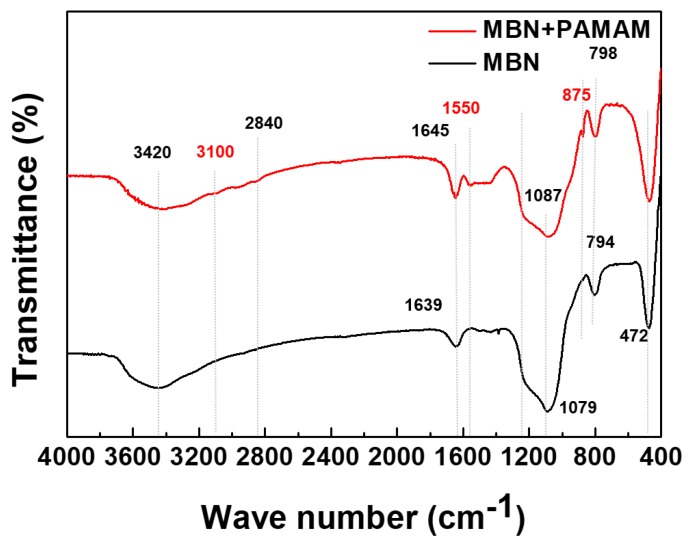
Fourier Transform Infrared Spectroscopy (FT-IR) spectra of MBN and PAMAM@MBN.

The chemical structures of MBN and PAMAN@MBN were examined through FT-IR spectroscopy (Figure 2). In the FT-IR spectra of MBN, a Si–O–Si asymmetric stretching and rocking vibration was detected at 794 and 472 cm^−1^ [42]. A peak at around 1079 cm^−1^ was made by the asymmetric stretching mode of Si–O–Si [42]. A peak at around 1639 cm^−1^ was caused by the water absorption of OH on its surface [43]. Also, a peak at around 3420 cm^−1^ was created by OH vibrations [42].

The FT-IR spectra of PAMAM@MBN was similar to MBN. A peak at 3100 cm^−1^ was related to the stretching bond of C–H. The band at 1550 cm^−1^ was due to either C–N amide stretching (amide II bands) [44] or a N–H-bending peak [45].

#### 3.1.3. Surface Area and Pore Size Analysis

**Figure 3:**
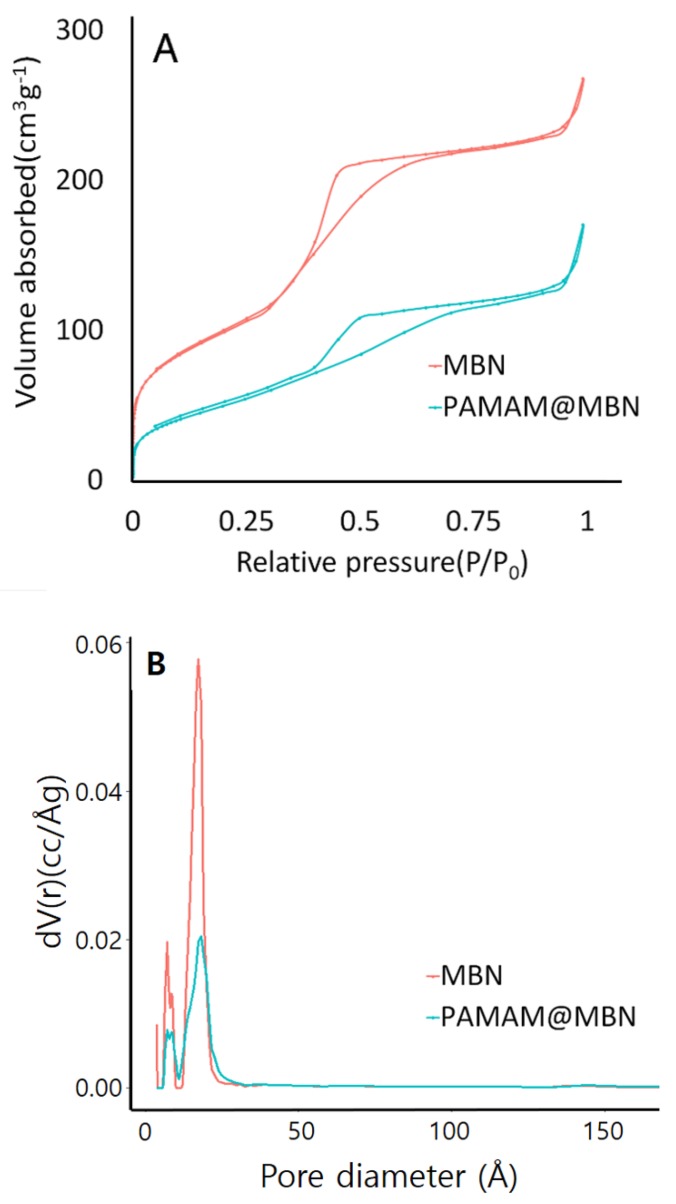
Nitrogen adsorption–desorption isotherms of MBN and PAMAM@MBN (**A**), and pore size distribution (**B**).

To assess the porosity, N_2_ adsorption–desorption isotherms were used. In Figure 3A, MBN and PAMAM@MBN exhibited characteristics of type IV isotherms of mesoporous materials, according to the IUPAC classification [46]. A type IV isotherm appears when a capillary tube is condensed by pore structures. Both MBN and PAMAM@MBN showed features of a type-H1 hysteresis loop. This is related to porous materials with relatively uniform pores. Table 1 shows the specific surface area (S_BET_), pore volume (V_p_), and pore diameter (D_p_) results of the BET and BJH methods. The pore size of MBN was 23.18 nm, which was similar to that of PAMAM@MBN, which had pores of 28.01 nm. The specific surface area in MBN was 358.45 m^2^/g, which was significantly reduced to 188.30 m^2^/g in PAMAM@MBN. This showed that the micropores of MBN was filled with PAMAM molecules. In addition, the total pore volume in MBN was 0.41 cc/g, and it was 0.26 cc/g in PAMAM@MBN, which was sharply reduced. The pore size distribution was similar between MBN and PAMAM@MBN, but the frequency of the distribution was clearly different (Figure 3B). The pore sizes of both MBN and PAMAM@MBN showed a narrow distribution that was close to 23.18 and 28.01 nm. With regard to the frequency of the pore distribution, MBN had more pores that were close to the average compared to PAMAM@MBN.

#### 3.1.4. FESEM and FETEM

**Figure 4:**
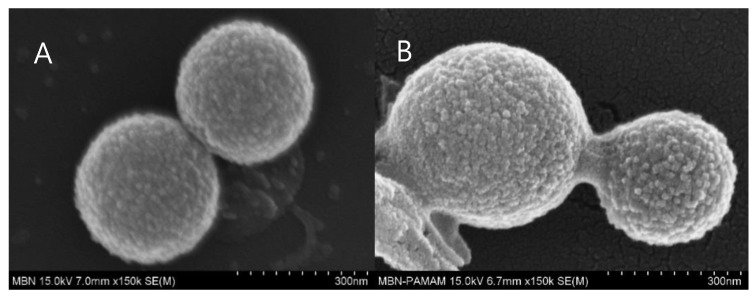
Analytical field-emission scanning electron microscopy (FESEM) images of (**A**) MBN and (**B**) PAMAM@MBN.

**Figure 5:**
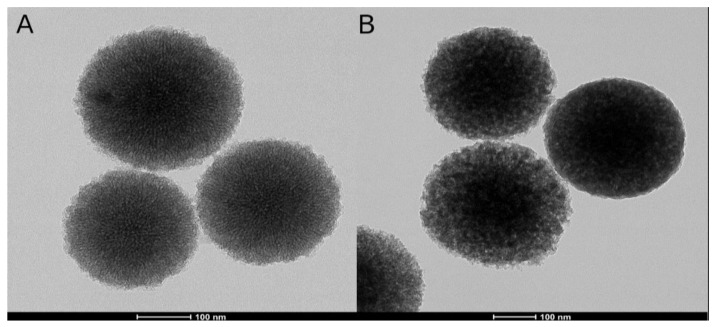
Images (200 kV Field-Emission Transmission Electron Microscopy (FETEM)) of (**A**) MBN and (**B**) PAMAM@MBN.

The forms of the samples were identified with FESEM and FETEM. The MBN and PAMAM@MBN molecules were 250–350 nm in diameter (Figure 4). With images from FESEM and FETEM, sphere-shaped MBN molecules were observed. In PAMAM@MBN, the MBN molecules were coated with PAMAM (Figure 5).

#### 3.1.5. In Vitro Ion Dissolution Test

**Figure 6:**
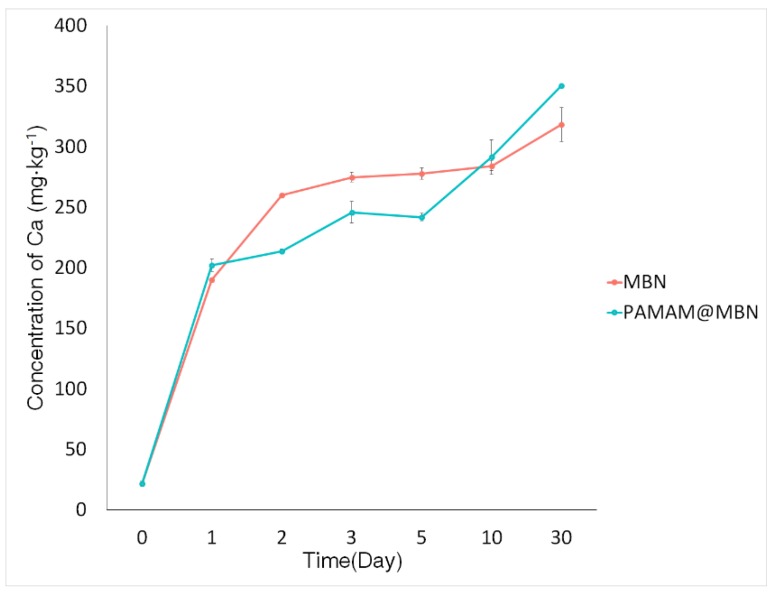
Calcium ion’s concentration over time.

The ion-releasing abilities of MBN and PAMAM@MBN are shown in Figure 6. The calcium ion levels in MBN and PAMAM@MBN sharply increased to 200 ppm after 24 h. Mesoporous bioactive glass nanoparticles showed a slight increase in levels from 2 to 30 days, but the increase was not very large. However, the volume of calcium ions in PAMAM@MBN showed a small increase of up to 3 days, then it decreased from 3 to 5 days, and finally resumed its increase from 5 to 30 days.

### 3.2. In Vitro Mineralization Ability Test

**Figure 7:**
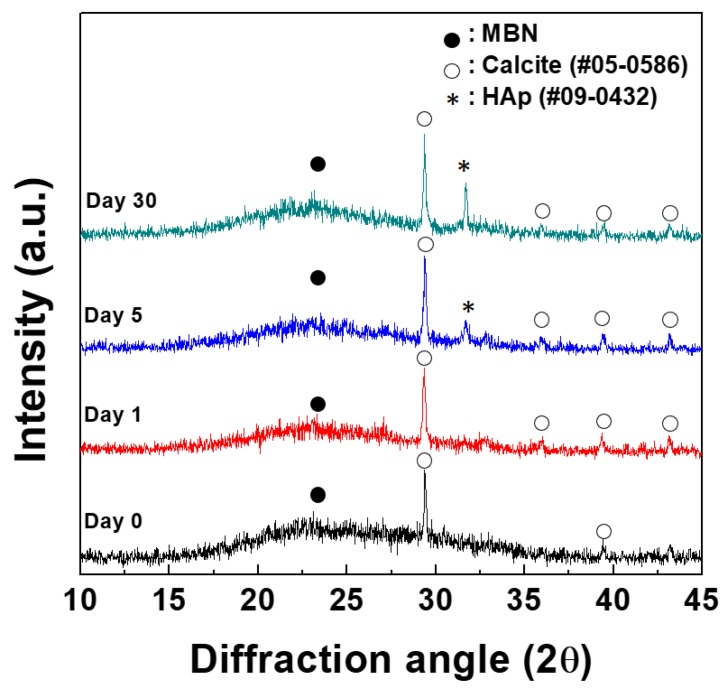
X-ray diffraction patterns of PAMAM@MBN at 1, 5, and 30 days after soaking in simulated body fluid (SBF) solution.

Figure 7 shows the XRD patterns of PAMAM@MBN after soaking in SBF solution for 30 days. Amorphous and crystalline phase peaks in the 2θ range of 15° to 30° were detected for residual MBN (amorphous phase) and calcite (CaCO_3_, JCPDS #05-0586) after soaking in SBF. Any differences in the peak intensity and crystalline phase of the residual MBN and calcite were not observed after the soaking incubations. A newly observed XRD peak with low crystallinity appeared after soaking MBN@PAMAM in SBF for 5 days. Also, this XRD peak was determined to be the hydroxyapatite phase (HAp, Ca_10_(PO_4_)_6_(OH)_2_, JCPDS #09-0432) according to the XRD results of other studies. The height of the HAp peak (*) intensity increased from 5 days to 30 days.

**Figure 8:**
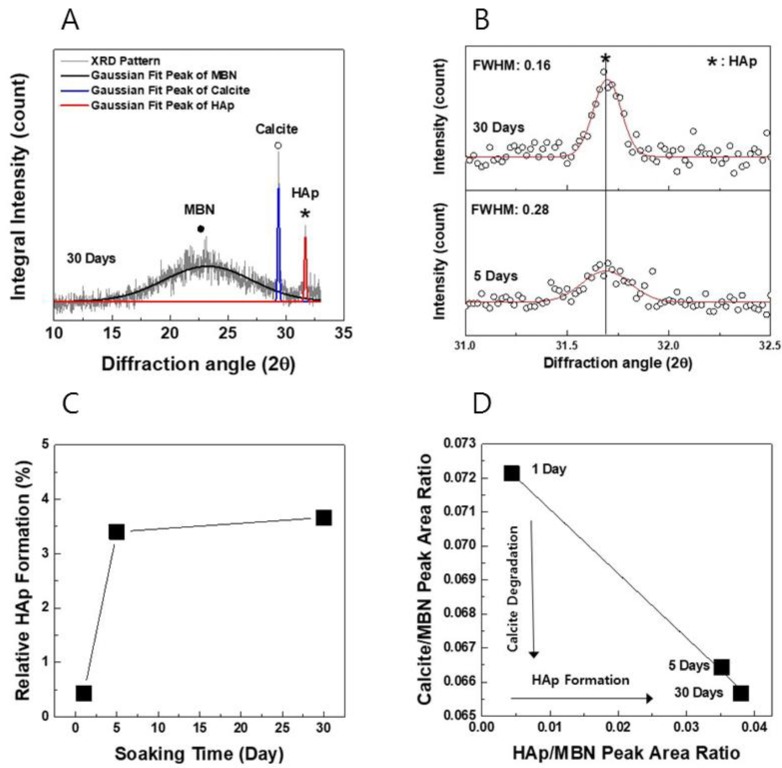
X-ray diffraction analysis and HAp formation of MBN@PAMAM after various soaking times. (**A**) XRD peak-fitting results of MBN@PAMAM with SBF after 30 days, (**B**) the full-width-half-maximum (FWHM) bandwidth of the XRD peak of the newly formed-HAp after 5 and 30 days, (**C**) the relative HAp formation rate over time, (**D**) the correlation between HAp formation and the degradation of calcite over time.

Figure 8 illustrates the XRD analysis and apatite-forming ability that was obtained after the interaction of MBN@PAMAM with SBF at various times. Figure 8A presents the pseudo-Gaussian peak-fitting results of the XRD pattern of MBN@PAMAM with SBF after 30 days. X-ray diffraction peak-fitting results were analyzed from the main peaks of amorphous MBN, calcite, and the newly formed-HAp crystalline phase. Figure 8B shows the full-width-half-maximum (FWHM) bandwidth of the XRD peak determined by curve-fitting the main XRD peak of the newly formed-HAp after 5 and 30 days. As shown in Figure 8B, the XRD peak intensity and crystallinity of the 30-day-incubated sample was found to be improved. Nevertheless, the 5-day sample showed no significant difference in the relative HAp formation rate, compared to the 30-day sample, from the results in Figure 8C. Figure 8D shows the correlation between HAp formation and the degradation of calcite in the interaction of MBN@PAMAM with SBF. Overall, it was found that the HAp formation of MBN@PAMAM increased with the effect of the degradation behavior of calcite in SBF.

**Figure 9:**
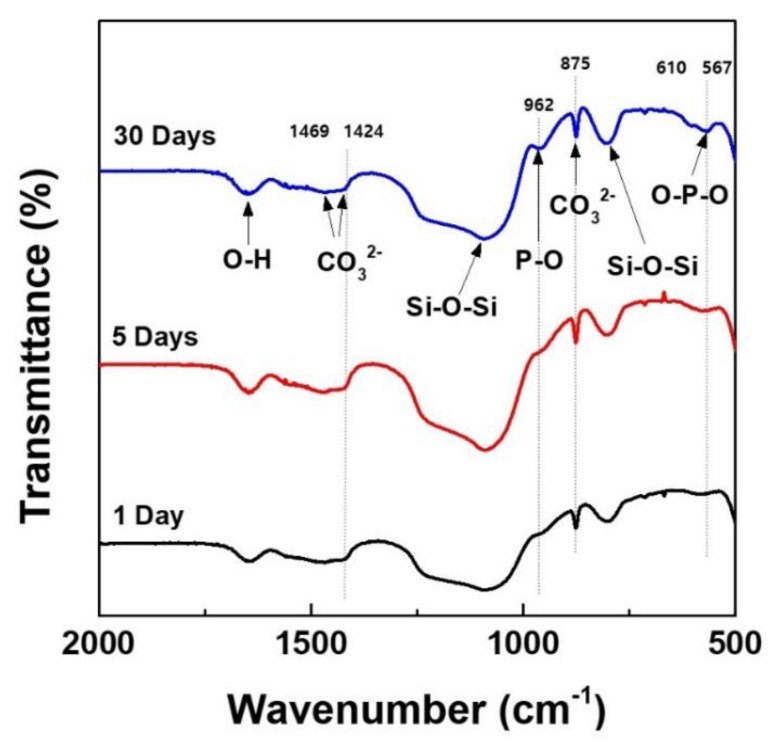
Fourier Transform Infrared Spectroscopy (FT-IR) spectra of MBN@PAMAM with SBF solution after various soaking times.

In order to confirm the apatite-forming ability of MBN@PAMAM, the samples were characterized by FT-IR spectroscopy. The IR spectra of the samples at different soaking times in SBF are shown in Figure 9. After immersing the samples in SBF for 30 days, the overall FT-IR spectra appeared to have five modes, corresponding to the characteristic vibration modes of the hydroxyl, silicate, phosphate, and carbonate groups. The bands at 567 cm^−1^ and 610 cm^−1^ were assigned to the O–P–O bending mode of the phosphate groups [47]. The band at 962 cm^−1^ was assigned to the symmetric stretching vibration mode of P–O bonding of phosphates group [48]. These FT-IR results show that the increase of the absorption bands in the phosphate group of the sample after 30 days was associated with the formation of the HAp phase, as shown in Figure 9. These results are also similarly in good agreement with the XRD results of Figure 7 and Figure 8. Furthermore, the formation of HAp was confirmed by observing the presence of the characteristic vibration modes of the carbonate group at 875, 1469, and 1424 cm^−1^, due to the carbonated hydroxyapatite (HCA) in the FTIR spectra [49,50]. Therefore, the apatite-forming ability of MBN@PAMAM could also be considered to be affected by the synergistic effect of bioactive MBN@PAMAM, and the degradation of the carbonate of calcite.

### 3.3. Comparison of Dentinal Tubule Occlusion

Table 2 shows the surface area ratio of the occluded dentinal tubules in each group. There was a statistically significant difference between the two groups (*p* < 0.05). The PAMAM@MBN group had the highest ratio of the occluded surface area. The two sets of the surface area ratio in each groups were compared with paired *t*-test, and method error was calculated with Dahlberg formula. No significant systematic error was found between the measurement ratio (*p* > 0.05), and the Dahlberg error was 1.10 [51].

**Figure 10:**
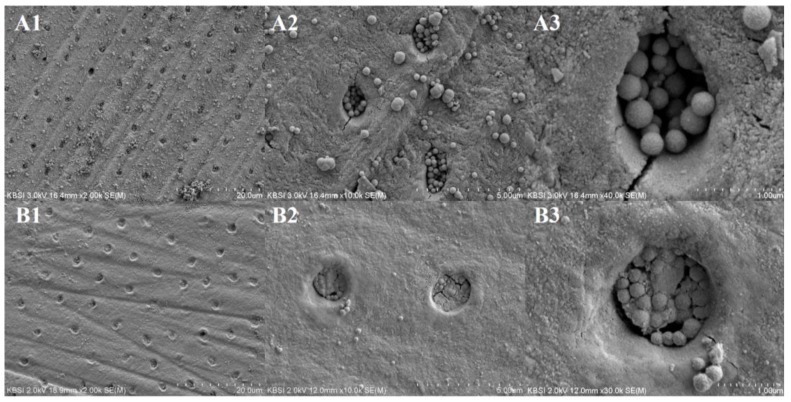
Field-emission scanning electron microscopy images (2000, 10,000, 40,000×) of the top sides of the samples that were used to determine the tubule-occluding effects in each group ((**A1**–**A3**) from MBN, and (**B1**–**B3**) from PAMAM@MBN).

Both the MBN and PAMAM@MBN groups sealed the dentinal tubules properly. However, there was little difference in the sealing pattern. The PAMAM@MBN showed perfect space sealing among the mesoporous bioactive glass particles. Figure 10(A2,A3) shows the space between the MBN particles in the dentinal tubule.

## 4. Discussion

This study aimed to enhance treatment efficiency by promoting each advantage of a highly reactive mesoporous bioactive glass that was introduced to reduce DH and the PAMAM dendrimer polymer. Regarding the reactivity of BAG, we could examine the size of the particle itself, because the smaller the particles, the more accelerated the potential formation of hydroxyapatite, as the biocompatibility, bioactivity [52], stability [53], and dissolution rate [54] improve [55]. According to the study of Fernando et al., it is known that nano-BAG powder forms a hydroxyapatite much faster [56]. In recent studies, mesoporous bioactive nanoparticles (MBNs), which have both the merits of particle size and porosity, have been developed, raising the possibility for the reconstruction of human hard tissue, including dental and bone parts, through the formation of apatites with calcium binding [36,57]. It is widely known that MBN in a spherical shape is more reactive and more efficient for dentin mineralization [14]. The mesoporous silica nanoparticles (MSN) were 100–350 nm in diameter, and the dentinal tubules were reported to be filled up to 3–4 µm deeper than with other DH treatment materials.

However, the particles were round, creating a gap between themselves, so the dentinal tubules were not completely closed against the tubule walls [43]. This could be a weakness of initial dentinal tubule sealing, and the improvement of patient discomfort. Poly(amidoamine) is a chemical polymer that has been utilized as a drug carrier, with various functional groups and a high utilization rate [27,28,29,30]. Using chemical polymers to link and to fill the gaps in MBN, pebbles of cement would be effective in closing the initial dentinal tubule. This study synthesized spherical MBN in a sol–gel method, and properly mixed it with PAMAM. Mixed PAMAM@MBN were found to be successfully synthesized, according to the results from XRD FTIR, FESEM, FETEM, and BET (Figure 1, Figure 2, Figure 3, Figure 4 and Figure 5).

The PAMAM@MBN synthesis process did not greatly change the characteristics of MBN. Through XRD and FTIR, it was found that calcium carbonate was created during the PAMAM and MBN synthesis processes (Figure 1 and Figure 2).

The formation of calcium carbonate separates the calcium ion from the polymer while the mineral is created. With FESEM and FETEM, only shapes could be examined. Thus, we used the BET method for more detailed examination. The MBN and PAMAM@MBN showed the same type of IV isotherm. The pore size of MBN was 23.18 nm, which was similar to PAMAM@MBN, with a pore size of 28.01 nm. The specific surface area of MBN was 358.45 m^2^/g, which was significantly reduced to 188.30 m^2^/g in PAMAM@MBN (Table 1). This shows that MBN pores were filled with PAMAM molecules. In addition, the total pore volumes of MBN were 0.41 cc/g, which was greatly reduced to 0.26 cc/g in PAMAM@MBN. Their pore-size distributions were similar, but they showed distinct differences in the distribution frequency. Nonetheless, MBN and PAMAM@MBN were similar in their ion-releasing abilities. In other words, the calcium ion levels increased in both MBN and PAMAM@MBN by up to about 200 ppm, 24 h later. The levels in MBN increased from 2 to 30 days, but the increases were small. The calcium ion-releasing ability of PAMAM@MBN increased slightly for up to 3 days, but then reduced from 3 to 5 days, and then began to increase again from 5 to 30 days. The PAMAM coating did not negatively affect the MBN ion-releasing ability (Figure 6).

An in vitro mineralization test was conducted to test the PAMAM@MBN mineralization effects. Pellet-shaped PAMAM@MBN samples 5 mm in diameter and 2 mm in thickness were incubated in SBF solution, and then dried after 1, 5, or 30 days, and then they were tested with XRD and FT-IR.

Consequently, after a day of soaking in SBF solution, a low-intensity hydroxyapatite peak began to appear, and it was continuously present from 5 to 30 days later. The FT-IR spectrum reflected an O–P–O banding mode (phosphate) at 567 cm^−1^ and 610 cm^−1^ [50], and the symmetric stretching vibration mode of P–O bonding (phosphates group) at 962 cm^−1^ [47] is related to hydroxyapatite formation 1 day later. This also suggests that each absorption band increased after 30 days, showing the apatite-forming ability of PAMAM@MBN, according to the XRD results (Figure 8). This demonstrates that PAMAM@MBN could be applied in DH treatment.

The key point of the DH treatment is to desensitize dentin through the remineralization of exposed dentin-occluding dentinal tubules [5,6].

To confirm the occluding effects of the dentinal tubules, changes in the shapes of the dentinal tubules were assessed with FESEM images after a desensitizing treatment was conducted on each of them.

In this study, there were statistically significant differences between the two groups (*p* < 0.05) (Table 2). The PAMAM@MBN groups had a higher ratio of occluded surface area. However, both the MBN and PAMAM@MBN groups sealed the dentinal tubules properly. There was also little difference in the sealing pattern. The PAMAM@MBN showed perfect sealing in the space between the mesoporous bioactive glass particles. Figure 10(A2,A3) shows the spaces between the MBN particles in the dentinal tubule.

As a method of DH treatment, research on bioactive glass has long been conducted [39,40]. Bioactive glass induces remineralization [24] on the external dentin disc, for the mechanical occlusion of the dentinal tubule, by releasing ions and developing a hydroxycarbonate layer [39]. However, it is hard to quickly deal with initial DH, as the mineralization activity is time-dependent. Dentin hypersensitivity is highly likely to be improved, if the initial effects of occluding dentinal tubules are good, as in the case of PAMAM@MBN. Therefore, the dentinal tubules are occluded in the initial stages, as with PAMAM@MBN; this is highly likely to improve the condition of DH. Based on the occluding test and the FESEM images of the dentinal tubules, the dentinal tubules treated by PAMAM@MBN were better sealed, because PAMAM filled the gaps between the MBN pores in PAMAM@MBN.

Bioactive glass is widely known to induce remineralization by releasing calcium, phosphate, silanol, and sodium ions. If the particles of bioactive glass are small and porous, its surface area becomes larger, which quickens the remineralization speed [14]. However, all MBN coated with PAMAM, which was used in the study, showed decreases in their specific surface areas and total pore volumes, but this positively affected the mineralization of PAMAM@MBN, according to the XRD and FTIR results, which demonstrated the mineral formation and ion-release processes.

In conclusion, PAMAM@MBN confirmed its capability for remineralizing, by successfully sealing exposed dentinal tubules. The MBN sealed the dentinal tubules, but it took time for it to improve the symptoms, as hydroxyapatite layers were created, which resulted in incomplete sealing. However, in this study, with the sealing effects of PAMAM, PAMAM@MBN is an efficient mixture for improving initial symptoms, and it would be further effective in dentinal tubule sealing and remineralization.

## 5. Conclusions

The study utilized PAMAM and MBN to promote treatment effects from the initial stages by effectively closing the dentinal tubules. The PAMAM@MBN was found to be successfully improved, according to results from XRD, FT-IR, FESEM, FETEM, and BET methods. It was identified that the PAMAM coating did not disrupt calcium ion release in MBN. It was also confirmed that minerals were created in a bioactivity test with soaking in SBF. Therefore, our study shows that PAMAM@MSN is an effective material for dentinal tubule sealing and remineralization.

## Figures and Tables

**Table 1:** Nitrogen adsorption results: specific surface area (S_BET_), pore volume (V_p_), and pore diameter (D_p_).

Samples	S_BET_ (m^2^/g)	V_p_ (cm^3^/g)	D_p_ (nm)
MBN	358.45 ± 10.75	0.41 ± 0.02	23.12 ± 1.39
PAMAM@MBN	188.30 ± 5.66	0.26 ± 0.02	28.01 ± 1.68

**Table 2:** Surface area ratio of the occluded dentinal tubule.

Groups	Occluded Area (%)	*p*-Value
MBN	80.35 ± 8.89	0.003
PAMAM@MBN	87.86 ± 5.62	
